# Leprosy and cutaneous leishmaniasis affecting the same individuals: A retrospective cohort analysis in a hyperendemic area in Brazil

**DOI:** 10.1371/journal.pntd.0010035

**Published:** 2021-12-13

**Authors:** Amanda Gabriela de Carvalho, Anuj Tiwari, João Gabriel Guimarães Luz, Daan Nieboer, Peter Steinmann, Jan Hendrik Richardus, Eliane Ignotti

**Affiliations:** 1 School of Medicine, Faculty of Health Sciences, Federal University of Rondonópolis, Rondonópolis, Mato Grosso, Brazil; 2 School of Medicine, Post-Graduation Program in Health Sciences, Federal University of Mato Grosso, Cuiabá, Mato Grosso, Brazil; 3 Department of Public Health, Erasmus MC, University Medical Center Rotterdam, Rotterdam, The Netherlands; 4 Swiss Tropical and Public Health Institute, Basel, Switzerland; 5 University of Basel, Basel, Switzerland; 6 School of Health Sciences, Post-Graduation Program Environment Sciences, State University of Mato Grosso, Cáceres, Mato Grosso, Brazil; University of California Davis, UNITED STATES

## Abstract

**Background:**

Leprosy and cutaneous leishmaniasis (CL) are neglected tropical diseases (NTDs) affecting the skin. Their control is challenging but the integration of skin NTDs control programs is recommended to improve timely detection and treatment. However, little is known about the occurrence of leprosy and CL in the same individuals, and what are the characteristics of such patients. This study aimed to identify and characterize patients diagnosed with both leprosy and CL (i.e., outcome) in the hyperendemic state of Mato Grosso, Brazil. Also, we investigated the demographic risk factors associated with the period between the diagnosis of both diseases.

**Methodology/principal findings:**

A retrospective cohort study was conducted with patients diagnosed between 2008 and 2017. From the leprosy (n = 28,204) and CL (n = 24,771) databases of the national reporting system, 414 (0.8%; 414/52,561) patients presenting both diseases were identified through a probabilistic linkage procedure. This observed number was much higher than the number of patients that would be expected by chance alone (n = 22). The spatial distribution of patients presenting the outcome was concentrated in the North and Northeast mesoregions of the state. Through survival analysis, we detected that the probability of a patient developing both diseases increased over time from 0.2% in the first year to 1.0% within seven years. Further, using a Cox model we identified male sex (HR: 2.3; 95% CI: 1.7–2.9) and low schooling level (HR: 1.5; 95% CI: 1.2–1.9) as positively associated with the outcome. Furthermore, the hazard of developing the outcome was higher among individuals aged 40–55 years.

**Conclusions/significance:**

Leprosy and CL are affecting the same individuals in the area. Integration of control policies for both diseases will help to efficiently cover such patients. Measures should be focused on timely diagnosis by following-up patients diagnosed with CL, active case detection, and training of health professionals.

## Author summary

Leprosy and cutaneous leishmaniasis (CL) are neglected tropical diseases (NTDs) that usually affect poor populations in the same geographical areas. Both affect the skin and can cause physical disability and disfigurement resulting in discrimination. Both diseases occur at hyperendemic levels in several regions of Brazil. The integration of skin NTDs control programs may be a way to improve timely detection and treatment. However, little is known about the occurrence of leprosy and CL in the same individuals, and what are the characteristics of these patients. For the Brazilian state of Mato Grosso, we identified 414 patients diagnosed with leprosy and CL between 2008 and 2017, spatially concentrated mainly in the North and Northeast mesoregions. The individual probability of developing both diseases increased from 0.2% to 1.0% within seven years. Male sex, being in the 40–55 years age group, and low levels of schooling were the risk factors positively associated with the time interval between the diagnosis of both diseases. These findings may inform the implementation of integrated leprosy and CL control policies focused on timely diagnosis. A sustainable integration requires continuous measures, such as the follow-up of patients, active case detection, training of health professionals, besides financial and political support.

## Introduction

Leprosy and cutaneous leishmaniasis (CL) are neglected tropical diseases (NTDs) affecting the skin [1]. They are responsible for approximately 0.2 [2] and 0.7–1.2 [3] million new cases worldwide every year. These diseases have many common clinical and epidemiological characteristics, but their causative agents are different. Leprosy is caused by *Mycobacterium leprae*, whereas CL is caused by the protozoan *Leishmania* parasites that are transmitted by the bite of phlebotomine sand flies. Both infections involve mucocutaneous tissues and produce a chronic granulomatous inflammatory response [4,5]. Both diseases are also a leading cause of deformity, stigma and discrimination in endemic populations [6]. The individuals experience negative psychosocial and economic impacts including reduced opportunities for education, employment, and social participation [6,7,8,9].

The key control strategy for leprosy and CL is early detection followed by an appropriate treatment, which is also necessary to prevent complications and deformities. For leprosy, the strategy has not managed to interrupt transmission, which has been relatively stagnant for more than a decade [2]. Many endemic countries are still struggling to achieve timely diagnosis, often due to control programs relying on passive case detection, weak health care delivery systems, and limited funding [1,10,11,12]. As a way to overcome these challenges, integration of vertical NTDs control programs and mainstreaming into primary health care is recommended to achieve cost-effectiveness, sustainability, and universal coverage [1,7,13]. Integration is deemed feasible because both diseases target lower socioeconomic strata of the population living in common geographical areas in the endemic countries. Such integration of skin NTDs programs has already been tested in some regions of Africa [1] and successful experiences have been reported [14].

Brazil is heavily burdened by leprosy and CL, globally and within the Americas [3,15]. The new case burden of leprosy is second after India [2]. An average of respectively more than 28,000 and 21,000 new cases of leprosy and CL are annually reported nationwide [15,16]. Historically, the highest detection rates are concentrated in states located in the North, Northeast, and Central-Western regions of the country. The central state of Mato Grosso is classified as a hyperendemic area for both leprosy and CL; it ranks first for leprosy and third for CL new cases in the country [15,17]. One third of the population of the state of Mato Grosso lives in municipalities classified as high risk for both diseases [18]. Despite this situation, no current measures aim at the integrated control of these diseases.

The first step towards program integration is the identification of endemic areas with geographical overlap [7]. It is also relevant to know whether leprosy and CL are affecting the same patients as well as the characteristics of these patients. Understanding the dynamics of occurrence at the individual level is crucial for designing and implementing integrated control policies. Therefore, the current study aimed to identify and characterize patients diagnosed with both leprosy and CL in the Brazilian state of Mato Grosso. In addition, we investigated demographic risk factors that could be associated with the time interval between the diagnosis of both diseases in the same individuals.

## Methodology

### Ethics statement

This study was approved by the Ethical Committee for Human Research of the Federal University of Rondonópolis (CAAE number 01735018.6.0000.8088). As all information was obtained from secondary data sources, the participants’ consent was not required.

### Study setting

The state of Mato Grosso is located in the Southern Amazon region in Central-Western Brazil. It is composed of 141 municipalities with 3,526,220 inhabitants [19]. The case definition of leprosy and CL follows the guidelines of the Brazilian Ministry of Health. For leprosy, the diagnosis is mainly confirmed based on clinical evidences [20], whereas the diagnosis of CL may be confirmed by either clinical and epidemiological or laboratory criteria [21]. From 2008 to 2017, Mato Grosso reached hyperendemic levels of leprosy and CL, with a cumulative incidence of 89.4 and 79.1 cases/100,000 inhabitants, respectively. Both diseases are heterogeneously distributed over the state, with an extensive area of geographical overlap in the North and Northeast mesoregions [18].

### Study design

We followed a retrospective cohort study design based on secondary data. The main event of interest (i.e., outcome) was the diagnosis of leprosy or CL in patients from Mato Grosso previously diagnosed with either disease between 2008 and 2017. Individuals presenting the outcome were identified through a probabilistic linkage procedure and their spatial distribution was mapped based on the location data in their health records. Next, we estimated the individual probability of developing the outcome by survival analysis and investigated associations between demographic risk factors and the time until the outcome using the Cox proportional hazards method.

### Data collection and study population

In Brazil, both leprosy and CL are notifiable diseases. Therefore, all cases should be reported to the Brazilian Notifiable Diseases Information System (SINAN–*Sistema de Informação de Agravos de Notificação*). A report triggers clinical and epidemiological follow-up, which facilitates diagnosis, medication supply, and surveillance [15,16]. We collated data on the occurrence of leprosy and CL from SINAN which in Mato Grosso is maintained by the Epidemiological Surveillance Sector of the Health Department of the state of Mato Grosso. As criteria, we included all new leprosy and CL cases reported in the state between January 1, 2008 and December 31, 2017. Further, we excluded relapse cases, duplicate entries, individuals residing outside Mato Grosso, records with inconsistencies in the name of the patient, later entries indicating an error or change in the diagnosis, and other entries referring to modifications to the database rather than local new cases (i.e., transfers, unknown, cases reinserted in the system for a new treatment round after abandonment or therapeutic failure). For each patient, we recorded information on the following variables: patient’s name, mother’s name, date of diagnosis, health service where cases were reported, date of birth, municipality of residence, sex, race, schooling level, and residential area.

The population estimates and the municipal cartographic shape files were obtained from the Brazilian Institute of Geography and Statistics (IBGE–*Instituto Brasileiro de Geografia e Estatística*) (ftp://geoftp.ibge.gov.br/).

### Data management and analysis

#### Probabilistic linkage procedure

**Fig 1:**
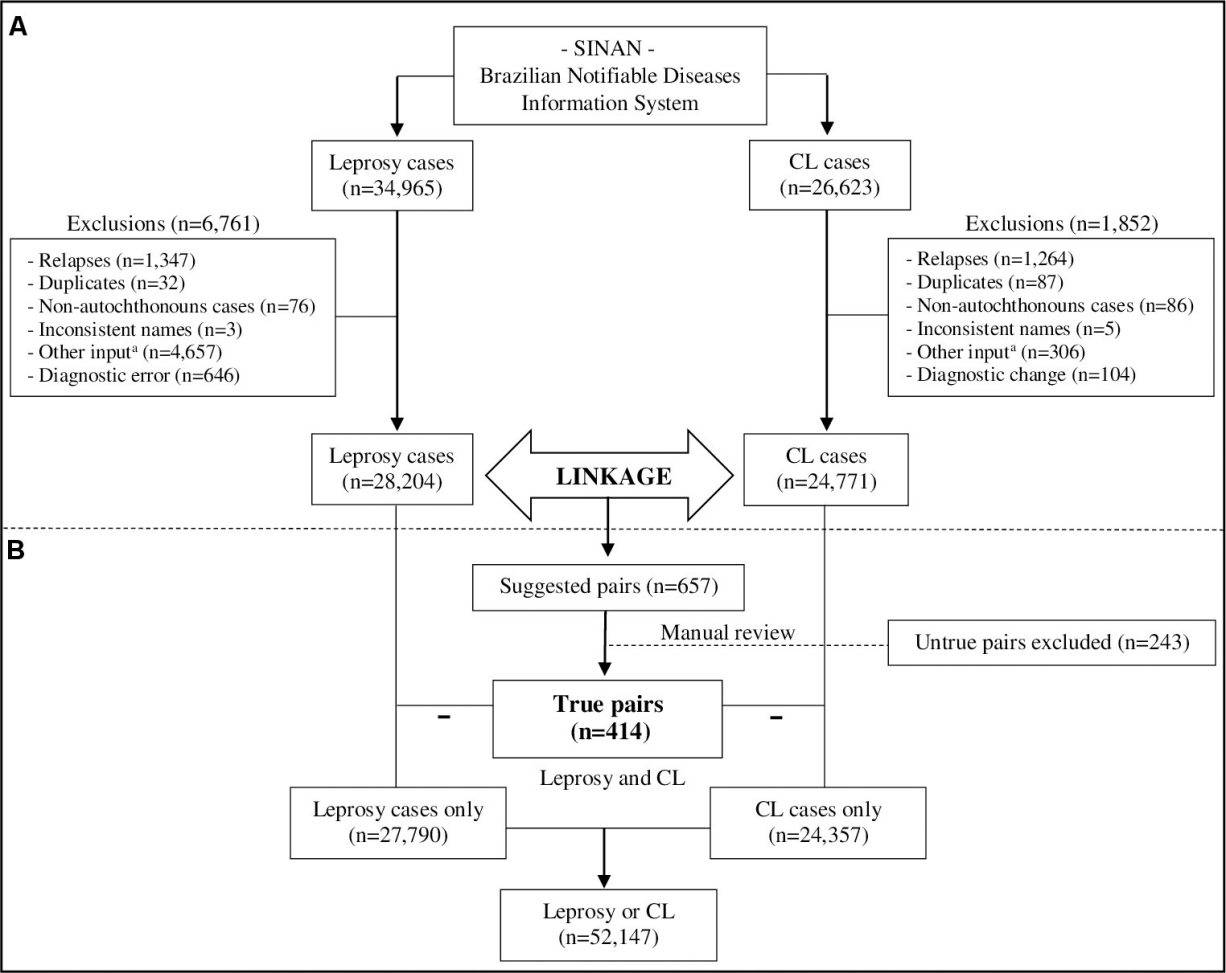
Identification of the study population. (A) Exclusion criteria. (B) Linkage between leprosy and cutaneous leishmaniasis (CL) datasets from the Brazilian Notifiable Diseases Information System (SINAN). Data from Mato Grosso state, Brazil, 2008–2017. ^a^ Transfers (within the municipality, from another municipality, from another state, or from another country), unknown, or cases reinserted in the system for a new treatment round after abandonment or therapeutic failure.

After applying the inclusion/exclusion criteria, leprosy (n = 28,204) and CL (n = 24,771) datasets were submitted to a direct probabilistic linkage procedure in Registry Plus Link Plus 3.0 beta software (Centers for Disease Control and Prevention, CDC, http://www.cdc.gov) (Fig 1A). This procedure consists of linking two or more datasets through common information, which allows the identification of individuals simultaneously present in two or more datasets [22].

The soundex code (i.e., the encoding of records by a phonetic system) of the patient’s name, sex, and date of birth was used in the blocking step. The blocking step consists of breaking records into blocks to reduce data processing time [23]. From leprosy and CL datasets, we matched patient’s name, sex, date of birth, mother’s name, and municipality of residence in pairs. For each pair, the software automatically calculates a score describing the probability of it being a true pair. The higher a score, the more likely it is to be a true pair. We adopted a cutoff point equal to seven to maximize the number of true matches, as recommended by the Brazilian Ministry of Health [23]. This enabled the formation of pairs with a maximum score of 30.8 and a bonding rate (i.e., number of true pairs/total number of formed pairs x 100) of 63.0%. All matched pairs were cross-checked manually to confirm correct matching. The correctly matched pairs (i.e., those patients diagnosed with both leprosy and CL) formed the outcome group. The other patients diagnosed with only one disease (leprosy or CL) represented the control group.

The observed number of patients in the outcome group was further compared with what would be theoretically expected in the study area if cases occurred randomly. Considering leprosy and CL as independent events, the expected number of patients diagnosed with both leprosy and CL over 10 years (2008–2017) was calculated by multiplying the reported case detection coefficients for each disease in the state of Mato Grosso [18] by the average population of the state (i.e., 3,150,671 individuals) in the same period. The population was averaged considering the official annual population estimates defined by the IBGE.

#### Spatial distribution

From the outcome group, the spatial distribution of individuals was analyzed considering their municipality of residence as analytical units. We mapped the absolute number of patients within each municipality of the state of Mato Grosso and calculated the cumulative detection coefficients by dividing the total number of patients diagnosed with both diseases by the estimated population size. The result was expressed per 100,000 inhabitants. To investigate the spatial autocorrelation between municipal detection coefficients, we used the Global Moran’s Index (I). The index ranges from -1 (inverse correlation) to +1 (direct correlation) and values close to zero indicate random distribution. Further, we applied Local Indicators of Spatial Association (LISA) to identify the location of spatial clusters. Based on the Moran Scatter Diagram, LISA values were mapped according to the following quadrants: High-High (positive values, positive means), Low-Low (negative values, negative means), High-Low (positive values, negative means), and Low- High (negative values, positive means). The High-High and Low-Low quadrant municipalities represented municipalities with similar neighbors (concordant areas), whereas High-Low and Low-High represented municipalities with different neighbors (transition areas) [24]. High-High municipalities were considered hotspots for the occurrence of leprosy and CL in the same individual.

Both Moran’s analyses were performed in GeoDa 1.10 software using a queen contiguity spatial weights matrix based on first order neighbor. The significance level at *p* < 0.05 was adopted for spatial autocorrelation. Mapping was conducted in the QGIS 3.4.0 software [25].

#### Survival analysis and Cox regression

To investigate the probability of developing the outcome over time, we estimated the survival curve using the Kaplan-Meier method considering the entire patient cohort (i.e., 52,561 individuals). For the outcome group, the survival time was calculated as the difference between the dates of diagnosis of the second and the first disease (in days). For the control group (patients right censored), the survival time was defined as the difference between the end date of the study and the date of diagnosis of leprosy or CL (in days). We estimated survival curves considering the following demographic covariates: sex (male versus female); age (0–29 years versus ≥ 30 years); race (mixed versus non-mixed); years of schooling (children/teenagers versus 0–4 full years or, > 4 full years); and area of residence (urban versus rural). The dichotomization of the race variable into mixed and non-mixed was based on the high proportion (52.4%) of mixed individuals in the population of the State of Mato Grosso [26,27]. The log-rank test was employed to check for differences between groups regarding the time until the outcome. Differences with *p* < 0.05 were considered statistically significant. The variables such as age, race, years of schooling, and area of residence had missing values at a rate ranging from 1.3% to 9.9%. Therefore, we imputed them with a multiple imputation model that included the variables with missing data, outcome, sex, and cumulative hazard estimated by the Nelson-Aalen estimator and the outcome indicator. The stability of the results was checked by sensitivity analysis under different assumptions (S1 Table).

To model the time until the outcome as a function of demographic predictors, we applied Cox proportional hazards regression. Furthermore, in the analysis we considered plausible interactions of schooling with age and area of residence. The final adjusted model was developed by a backward stepwise procedure. We checked the effect of removing predictors on the model fit through likelihood ratio test (LRT) that compared nested models. Variables with *p* < 0.05 in the adjusted model were considered as associated with the time elapsed until the outcome. For both unadjusted and adjusted analysis, we calculated the hazard ratio (HR) with 95% confidence interval (CI) for each predictor.

The age was treated as a continuous variable and its linear behavior was verified by the Martingale residual plot of the null model. In our model, age was specified in linear and non-linear terms; the latter was adjusted by the P-spline function with three degrees of freedom. P-spline is a polynomial smoothing function that considers a set of regularly spaced knots and fit nonlinear data by creating continuous and flexible estimate of the predicted variable [28]. For the linear term, the results were presented as the HR calculated for the 75th percentiles versus the HR for the 25th percentile. The shape of non-linear associations was illustrated as log hazard curves with 95% CI.

The assumption of proportional hazards was evaluated by the Schoenfeld residual test and visual inspection of plots of the residuals over time. We also checked for the presence of outliers using the Martingale and Deviance residual plots versus the index number of observations. Furthermore, the effect of smoothing on the variable age was assessed by a LRT that compared the final model with a model containing the same explanatory variables without smoothing.

Survival analysis, multiple data imputation, and Cox model estimation were performed in R studio 3.6.2 [29] software using the *survival* [30], *mice* [31] and *smoothHR* [32] packages.

## Results

Through the probabilistic linkage procedure between leprosy and CL datasets, we identified 657 suggestive pairs of patients diagnosed with both diseases in the state of Mato Grosso from 2008 to 2017. Of these, 243 were identified as false matches after manual inspection, and treated as individuals with only one disease. Finally, 414 of 52,561 (0.8%; 95% CI: 0.7–0.9; 788/100,000) individuals with leprosy, CL or both diagnoses were included in the analysis (Fig 1B). This observed number was almost 19 times higher than the number of cases with both diagnoses that would be expected based on chance alone, i.e., 22 cases. As shown in Table 1, most of these individuals were first diagnosed with CL (57.2%; 95% CI: 52.5–62.0; 237/414). Most leprosy (83.8%; 95% CI: 80.3–87.4; 347/414) and CL (71.7%; 95% CI: 67.4–76.1; 297/414) cases were reported by primary healthcare services. Within one year, 26.8% (95% CI: 22.5–31.1; 111/414) of the patients were diagnosed with leprosy and CL, while 59.4% (95% CI: 54.7–64.1; 246/414) were diagnosed within three years. The average time for a patient who had received an initial diagnosis of leprosy to be also diagnosed with CL (and vice versa) was 2.9 years.

**Table 1:** Frequency distribution of patients diagnosed with leprosy and cutaneous leishmaniasis (CL) according to the time interval until the diagnosis of the second disease and the order of diagnosis. Data from Mato Grosso state, Brazil, 2008–2017.

Time interval (years)	First diagnosis				Total^a^^,^^b^		
	Leprosy		CL				
	n	% (95% CI)	n	% (95% CI)	n	% (95% CI)	%*
0 |– 1	42	10.2 (7.2–13.1)	69	16.6 (13.1–20.3)	111	26.8 (22.5–31.1)	26.8
1 |– 2	34	8.3 (5.6–10.9)	42	10.1 (7.2–13.1)	76	18.4 (14.6–22.1)	45.2
2 |– 3	32	7.7 (5.2–10.3)	27	6.5 (4.1–8.9)	59	14.2 (10.9–17.6)	59.4
3 |– 4	19	4.6 (2.6–6.6)	21	5.1 (3.0–7.2)	40	9.7 (6.8–12.5)	69.1
4 |– 5	23	5.6 (3.3–7.8)	24	5.8 (3.5–8.0)	47	11.4 (8.3–14.4)	80.5
5 |– 6	12	2.9 (1.3–4.5)	14	3.4 (1.6–5.1)	26	6.3 (3.9–8.6)	86.8
6 |– 7	3	0.7 (0.0–1.5)	14	3.4 (1.6–5.1)	17	4.1 (2.2–6.0)	90.9
7 |– 8	6	1.4 (0.3–2.6)	12	2.9 (1.3–4.5)	18	4.3 (2.4–6.3)	95.2
8 |– 9	6	1.4 (0.3–2.6)	13	3.2 (1.5–4.8)	19	4.6 (2.6–6.6)	99.8
9 |– 10	0	0.0 (0.0–0.0)	1	0.2 (0.0–0.7)	1	0.2 (0.0–0.7)	100.0
**Total**	177	42.8 (38.0–47.5)	237	57.2 (52.5–62.0)	**414**	**100.0**	-

^a^ Health services where leprosy cases were reported: primary healthcare centers (83.8%; 95% CI: 80.3–87.4; 347/414), hospitals (3.6%; 95% CI: 1.8–5.4; 15/414), specialized centers (6.8%; 95% CI: 4.3–9.2; 28/414) and others/unknown (5.8%; 95% CI: 3.5–8.0; 24/414).

^b^ Health services where CL cases were reported: primary healthcare centers (71.7%; 95% CI: 67.4–76.1; 297/414), hospitals (12.6%; 95% CI: 9.4–15.7; 52/414), specialized centers (5.8%; 95% CI: 3.5–8.0; 24/414) and others/unknown (9.9%; 95% CI: 7.0–12.8; 41/414).

%: relative frequency;

%*: cumulative frequency; CI: Confidence Interval.

**Fig 2:**
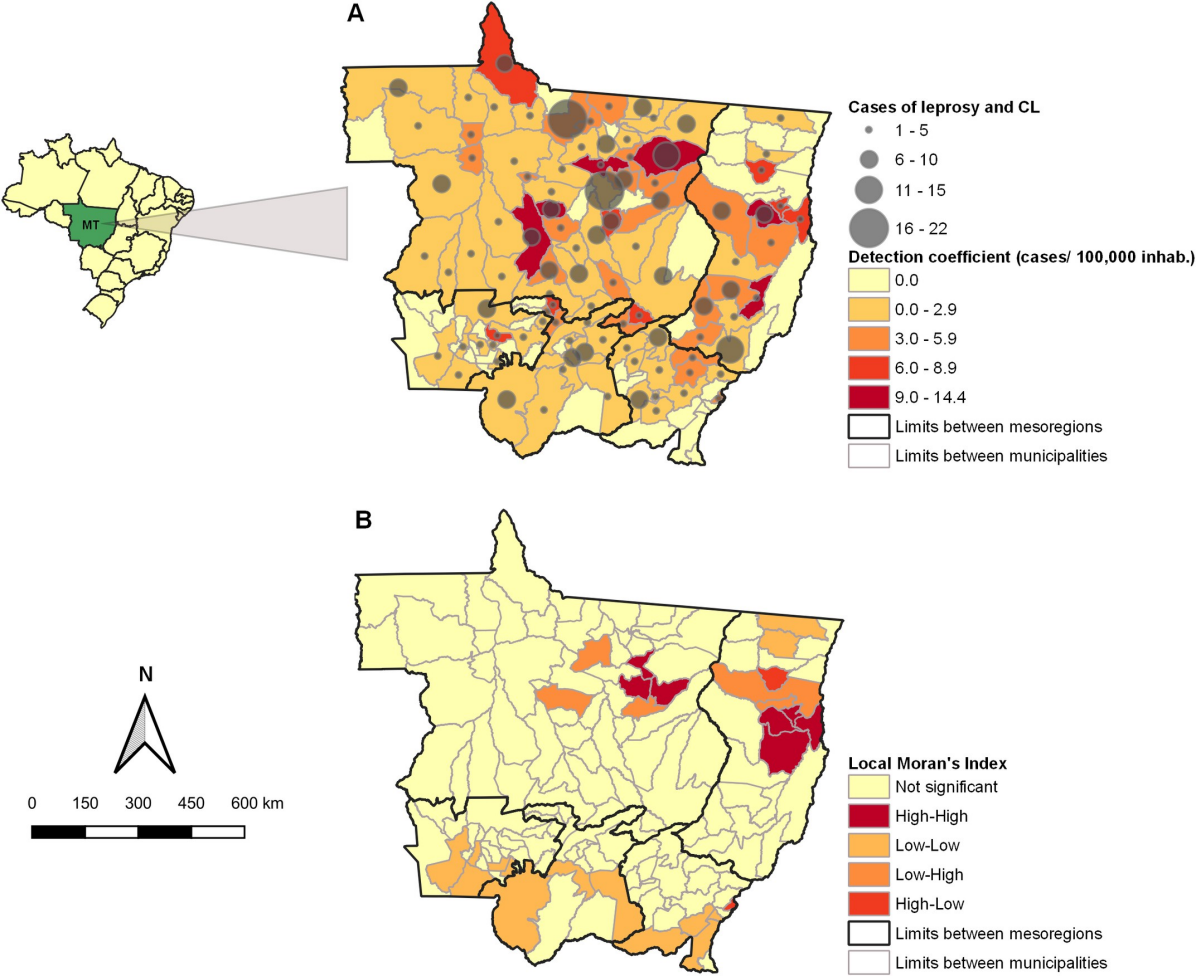
Geographic characterization of the patients diagnosed with leprosy and cutaneous leishmaniasis (CL) in Mato Grosso state, Brazil, 2008–2017. (A) Represents the absolute number of patients and the cumulative detection coefficient for leprosy and CL in the same individuals per municipality. (B) Represents the local Moran’s Index analysis for the cumulative detection coefficient per municipality. The digital georeferenced database of the municipalities was obtained from the Brazilian Institute of Geography and Statistics (https://geoftp.ibge.gov.br/organizacao_do_territorio/malhas_territoriais/malhas_municipais/municipio_2018/UFs/MT/MT.zip).

The mean age (standard deviation) of the individuals affected by both diseases was 43.9 (15.0) years. Regarding sex, race, schooling, and residential area, patients were predominantly male (83.1%; 95% CI: 79.5–86.7), mixed race (52.9%; 95% CI: 48.1–57.7), had a low level of schooling (55.3%; 95% CI: 50.5–60.1) and were urban residents (68.6%; 95% CI: 64.1–73.1) (S2 Table). The patients were distributed throughout 98 of the 141 municipalities of Mato Grosso, and the number of cases per municipality ranged from 0 to 22. In the outcome group, the highest cumulative detection coefficients were observed in municipalities located in the North and Northeast mesoregions with a peak of 14.4 cases/ 100,000 inhabitants (Fig 2A). In fact, the Moran’s analysis revealed a positive spatial autocorrelation of the cumulative detection coefficients per municipality (I = 0.171; *p* = 0.002) and hotspots for the occurrence of both diseases in the same individuals in the North and Northeast mesoregions (Fig 2B).

The Kaplan-Meier survival curve indicated that the cumulative probability of an existing patient to develop CL or leprosy was 0.2% (95% CI: 0.2–0.3), 0.5% (95% CI: 0.5–0.6) and 1.0% (95% CI: 1.0–1.1) within one, three, and seven years after the diagnosis of the first disease, respectively (S1A Fig). In addition, male patients, aged ≥ 30 years, of mixed race, and with low level of schooling (0–4 full years) had significantly shorter survival times when compared to female patients, < 30 years, non-mixed, and with more advanced schooling (> 4 full years), respectively (S1B, S1C, S1D and S1E Fig).

**Fig 3:**
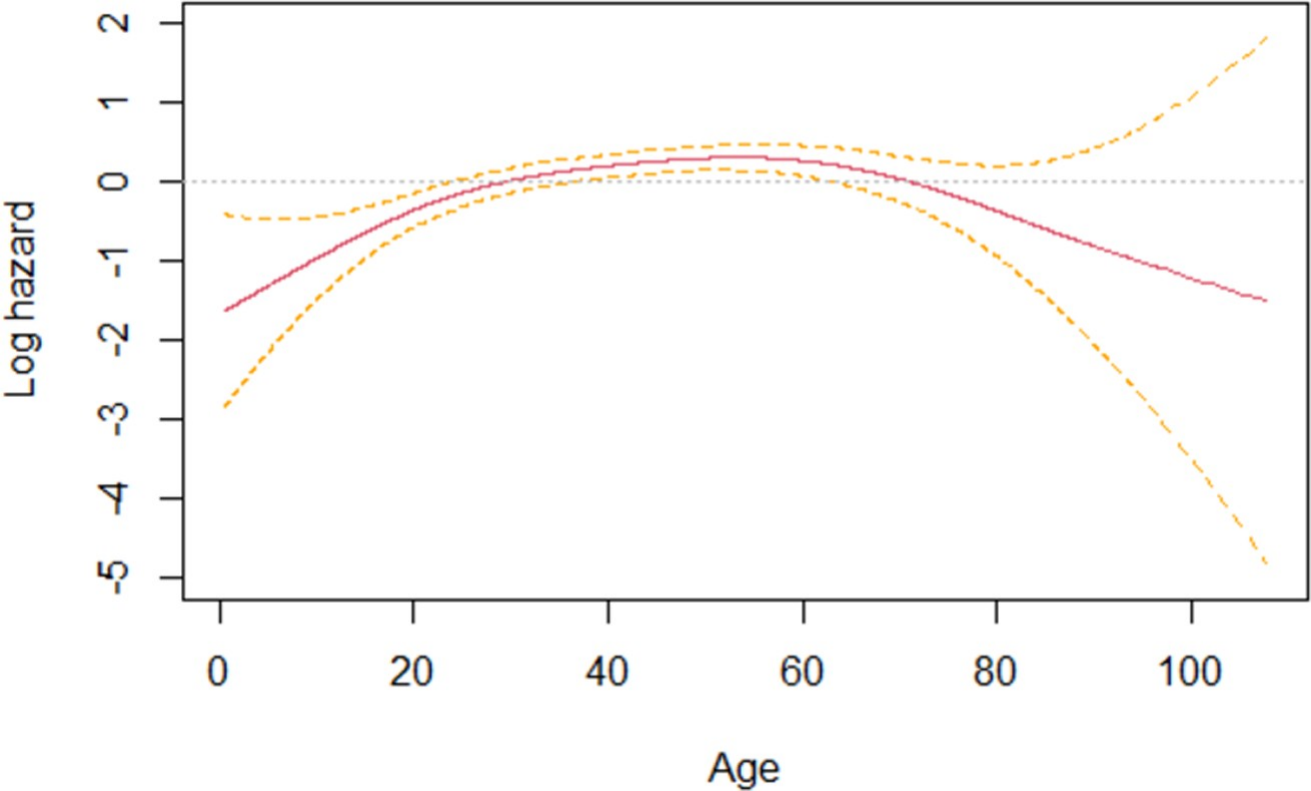
Effect of age on the time interval between the diagnosis of leprosy and cutaneous leishmaniasis in the same individuals according to Cox regression model with P-splines. Solid line represents spline coefficient of each estimated knot while dashed lines are the limits of the 95% confidence intervals. Data from Mato Grosso state, Brazil, 2008–2017.

Table 2 summarizes the estimated HR for demographic risk factors associated with the time interval until the diagnosis of the second disease (leprosy or CL) in both unadjusted and adjusted analyses. Given the adjusted model, the variables sex, schooling, and age (linear and non-linear terms) were significantly associated with the outcome. The hazard of males to present with both leprosy and CL was 2.3 (95% CI: 1.7–2.9) times higher when compared to females. Individuals with low schooling level were 1.5 (95% CI: 1.2–1.9) times more likely to be diagnosed with a second disease than individuals with better schooling. Taking the 25th percentile as the reference for the linear term of age, we observed that patients aged 52.5 years were at higher risk (HR: 1.5; 95% CI: 1.1–1.9) of being diagnosed with a second disease when compared to patients aged 27.0 years (Table 2). In Fig 3, the logarithmic risk curve smoothed with the P-spline function illustrates the curvilinear shape of the association between age (non-linear term) and the outcome. In agreement with the results of the linear term, the risk of developing the outcome significantly increased among individuals of the age group 40–55 years.

**Table 2:** Cox proportional hazards models for the time elapsed between the diagnosis of leprosy and cutaneous leishmaniasis in the same individuals. Data from Mato Grosso state, Brazil, 2008–2017.

	Unadjusted model^a^		Adjusted model^b^	
Variables	HR (95% CI)	*p—*value	HR (95% CI)	*p—*value
**Sex**				
Male	2.3 (1.8–2.9)	< 0.001*	2.3 (1.7–2.9)	< 0.001*
Female	1		1	
**Age (years)**				
75^th^ percentile vs. 25^th^ percentile^c^	1.6 (1.3–2.1)	< 0.001*	1.5 (1.1–1.9)	0.016*
**Race**				
Mixed	1.2 (1.0–1.5)	0.040*	-	-
Non-mixed^d^	1		-	
**Schooling (full years)**				
0–4	1.9 (1.5–2.3)	< 0.001*	1.5 (1.2–1.9)	< 0.001*
> 4	0.7 (0.6–0.8)	0.001*	-	-
Children/teenagers^e^	1		1	
**Residential area**				
Urban	1.0 (0.8–1.2)	0.800	-	-
Rural	1		-	

^a^ Univariate analysis.

^b^ Multivariate analysis: Cox proportional hazards regression model with smoothing P-splines.

^c^ 75th percentile: 52 years; 25th percentile: 27 years.

^d^ White, black, Asian, or indigenous.

^e^ Individuals aged < 18 years.

HR: Hazard Ratio; CI: Confidence Interval.

## Discussion

This is the first population-based study that identified and characterized patients with both leprosy and CL in a Brazilian hyperendemic area—the State of Mato Grosso. Patients presenting this outcome were heterogeneously distributed throughout the state. Sex, age group and level of schooling were the demographic indicators that were statistically significantly associated with the time elapsed between the diagnosis of these two diseases in the same individuals.

We identified a considerable number of patients who had been diagnosed with both leprosy and CL (n = 414, 0.8% of the cohort with either disease). This contrasts with the few reports about such associations in the scientific literature, and suggests that the topic has not been properly studied. In fact, to date, only case reports/cases series have been published [4,5,33,34]. The occurrence of leprosy and CL in the same patients does not seem to be a rare event, as we found many more cases with both diseases than expected if the distribution were truly random. Based on our findings, this is most likely due to common external/environmental risk factors. Both diseases are predominantly occurring in the same profile of patients, especially in the North and Northeast mesoregions of the state. This spatial distribution is consistent with a previous ecological study using patients diagnosed with only one of these diseases in the same timeframe [18]. In line with our results, Carvalho et al. [18] identified the overlapping of high-risk areas for the occurrence of leprosy and CL exactly in the North and Northeast of the state of Mato Grosso.

The probability of a patient being diagnosed with leprosy or CL increased every year given the previous diagnosis of one of these disease during our follow-up. It is possible that the occurrence of both diseases in the same patient has clinical implications. Studies addressing leprosy and geo-helminthiases have shown that infections by intestinal helminths may play an immunological role in the establishment of *M*. *leprae* infections, progression towards severe clinical forms, and the development of leprosy reactions [35,36]. Furthermore, Oliveira et al. [37] recently suggested that a co-infection by the protozoan *Toxoplasma gondii* has immunomodulatory properties that influence the susceptibility to leprosy. However, our current data do not support a hypothesis that leprosy is increasing the susceptibility to CL or the other way round. Thus, further studies should address this topic and investigate whether the presence of one disease influences clinical outcomes of another.

Given the long incubation period and the slow clinical progression of leprosy [12], it is reasonable to consider systematic leprosy screening for all CL patients seeking assistance in health facilities [5]. In fact, most patients who presented both diseases during our study period were first diagnosed with CL; within three years, most of them were diagnosed with the second disease. Thus, a follow-up of CL patients is particularly recommended to achieve early detection of leprosy cases among them. This could be achieved by integrating the vertical leprosy and CL control programs with an emphasis on timely diagnosis, as already proposed by Mitjá et al. [7]. To achieve success, it is important to ensure continuous training of professionals involved in primary healthcare services, such as community agents, nurses and physicians. The training should focus on identifying high risk groups for the next disease, based on the diagnostic history. Staff should be trained on differential diagnosis, basic dermatology, epidemiology, complication management, treatment and other important aspects for understanding the dynamics of the occurrence of each disease [7,14,38,39].

It is also desirable that the integration of control programs consider the grouping of diseases according to common sociodemographic characteristics [40]. For instance, school-aged children are already targeted for the integrated control of geo-helminthiasis as they have a high vulnerability to these infections [41]. In our study, males, individuals aged 40–55 years with low level of schooling were at highest risk of developing the second disease (leprosy or CL). This was consistent with the sociodemographic profile of leprosy and CL previously identified in the state of Mato Grosso [18].

The high risk of leprosy and CL found among males may be attributed to work-related activities and/or behavioral and cultural factors that exposed them more than females to the bite of infected sand flies and *M*. *leprae* infections [42,43]. Silva et al. [44] also described a higher number of leprosy cases among males than in females in the Brazilian state of Maranhão. For CL, the association between male sex and the occurrence of the disease is well established [42,45,46]. Guerra-Silveira & Abad-Franch [47] also observed that CL incidence rates are male-biased in the reproductive age in Brazil. In fact, our findings also suggested that adult individuals are more likely to present a second disease. In addition to a greater exposure to CL vectors [46], this increased risk may be attributed to the higher number of interpersonal contacts and the long incubation period of leprosy [12,44,48].

Similarly to others studies performed nationwide for leprosy [43] and CL [49], the detected relationship between low schooling levels and the occurrence of both diseases reinforces their label as neglected diseases of poverty. Given that schooling level is a proxy variable for socioeconomic status, it is likely that leprosy and CL associations are related to poor living conditions [50,51]. Thus, this association may enhance the well-known socioeconomic impact due to the stigma and physical disabilities often attributed to these diseases [8,52].

Even with the use of robust and integrated analytical approaches, the present study still has some limitations. First, the use of secondary data commonly implies missing information. As an attempt to fix that, we performed an imputation technique for missing data. Also, evidence of underreporting in SINAN has already been reported in Brazil [53]. However, given that both leprosy and CL reporting is mandatory for the supply of drugs for clinical treatment, we believe that the underreporting bias has a low impact on our results. In particular, it is noteworthy that the possibility of self-healing of skin lesions caused by *Leishmania braziliensis*—the predominant species in the study area [54], could lead to some underreporting of CL cases. In addition, our estimate may be conservative as we did not assess whether patients developed a second diseases outside the follow-up period. Finally, given the high occurrence of CL in rural/peri-urban areas, and given that CL clinical signs are more likely to be noticed than leprosy manifestations, it is likely that leprosy cases were underestimated as individuals from rural areas may have less access to health services capable of establishing a correct leprosy diagnosis.

Despite these limitations, our current findings, along with the previous identification of a geographical overlap of both diseases [18], strongly suggest that primary health care professionals should be aware that leprosy and CL may affect the same patients in hyperendemic areas. This awareness combined with active case detection plays a crucial role in timely diagnosis and treatment [55], which is the aim state- [56] and nationwide [57] for both diseases to decrease stigmatizing and other complications. The integration of control policies for leprosy and CL is a feasible way to achieve that [1]. Incidentally, other endemic skin NTDs may also be considered during the design of such policies [38,39]. For that, it is essential to ensure the provision of human and financial resources, as well as sustained political support through partnerships between the Ministry of Health, regional and local public health managers, training institutions and international bodies [7,14,40].

In conclusion, leprosy and CL are affecting the same individuals and the phenomenon is common in the Brazilian state of Mato Grosso. At-risk individuals are spatially concentrated in the North and Northeast mesoregions of the state. The probability of presenting a second diagnosis increased from 0.2% to 1.0% within seven years. Male sex, middle age and low levels of schooling were risk factors associated with the time interval between the diagnosis of the first and the second disease.

## Supporting information

S1 Table Sensitivity analysis under different assumptions to evaluate the consistency of the results presented in relation to the missing data imputation. Data from Mato Grosso state, Brazil, 2008–2017.
(DOCX)

S2 Table Frequency distribution of patients diagnosed with both leprosy and cutaneous leishmaniasis (CL) (outcome group) and patients diagnosed with only leprosy or CL (control group) according to demographic variables. Data from Mato Grosso state, Brazil, 2008–2017.
(DOCX)

S1 Fig Kaplan-Meier survival estimate for the time interval between the diagnosis of leprosy and cutaneous leishmaniasis in the same individuals. **(**A) Full cohort. (B) Sex. (C) Age group. (D) Race. (E) Schooling. (F) Residential area. The *p* values of the log-rank test were represented for each variable. Data from Mato Grosso state, Brazil, 2008–2017.
(TIF)

S1 STROBE Checklist STROBE Statement—Checklist of items that should be included in reports of *cohort studies*. (DOCX)
