# Supplementary material for: Leprosy and cutaneous leishmaniasis affecting the same individuals: A retrospective cohort analysis in a hyperendemic area in Brazil
Source: PLoS Negl Trop Dis. 2021 Dec 13;15(12):e0010035. doi: 10.1371/journal.pntd.0010035 (PMC8699965; doi:10.1371/journal.pntd.0010035)
Supplement: S1 Table — Data from Mato Grosso state, Brazil, 2008–2017. (DOCX) [file pntd.0010035.s001.docx]

**S1 Table. Sensitivity analysis under different assumptions to evaluate the consistency of the results**

**presented in relation to the missing data imputation.** Data from Mato Grosso state, Brazil, 2008-2017.

|  | **Model 1 ^a^** | | **Model 2 ^b^** | | **Model 3 ^c^** | |
| --- | --- | --- | --- | --- | --- | --- |
| **Variables** | **HR (95%CI)**  **adjusted** | ***p* - value** | **HR (95%CI)**  **adjusted** | ***p* - value** | **HR (95%CI)**  **adjusted** | ***p* - value** |
| **Sex** |  |  |  |  |  |  |
| Male | 2.3 (1.8-3.0) | < 0.001* | 2.3 (1.8-3.0) | < 0.001* | 2.2 (1.7-2.9) | < 0.001* |
| Female | 1 |  | 1 |  | 1 |  |
| **Age (years)** |  |  |  |  |  |  |
| 75^th^ percentile vs. 25^th^ percentile ^d^ | 1.5 (1.1-1.9) | **-** | 1.7 (1.3-2.2) | - | 1.4 (1.1-1.8) | - |
| **Race** |  |  |  |  |  |  |
| Mixed | - | - | 1.2 (1.0-1.5) | 0.053 | - | - |
| Non-mixed ^e^ | - |  | 1 |  | - |  |
| **Schooling (full years)** |  |  |  |  |  |  |
| 0-4 | 1.5 (1.2-1.9) | < 0.001* | - | - | 1.8 (1.5-2.2) | < 0.001* |
| > 4 | - | - | - | - | - | - |
| Children/teenagers ^f^ | 1 |  | - |  | 1 |  |

^a^ Assumption 1: all missing values were removed.

^b^ Assumption 2: all missing values were considered for the following categories: 0-4 years of schooling, mixed, urban and average age of 40 years.

^c^ Assumption 3: all missing values were considered for the following categories: > 4 years of schooling, non-mixed, rural and average age of 40 years.

^d^ 75th percentile: 52 years; 25th percentile: 27 years.

^e^ White, black, Asian or indigenous.

^f^ Individuals aged < 18 years old.

HR: Hazard Ratio; CI: Confidence Interval.
