# Supplementary material for: Leprosy and cutaneous leishmaniasis affecting the same individuals: A retrospective cohort analysis in a hyperendemic area in Brazil
Source: PLoS Negl Trop Dis. 2021 Dec 13;15(12):e0010035. doi: 10.1371/journal.pntd.0010035 (PMC8699965; doi:10.1371/journal.pntd.0010035)
Supplement: S2 Table — Data from Mato Grosso state, Brazil, 2008–2017. (DOCX) [file pntd.0010035.s002.docx]

**S2 Table. Frequency distribution of patients diagnosed with both leprosy and cutaneous leishmaniasis (CL) (outcome group) and patients diagnosed with only leprosy or CL (control group) according to demographic variables.** Data from Mato Grosso state, Brazil, 2008-2017.

| **Variables** | **Outcome group**  **(Leprosy and CL)** | | **Control group**  **(Leprosy or CL)** | |
| --- | --- | --- | --- | --- |
|  | **n** | **% (95% CI)** | **n** | **% (95% CI)** |
| **Sex** |  |  |  |  |
| Male | 344 | 83.1 (79.5-86.7) | 34,702 | 66.6 (66.1-67.0) |
| Female | 70 | 16.9 (13.3-20.5) | 17,445 | 33.4 (33.0-33.8) |
| **Age group (years)** |  |  |  |  |
| 0-29 | 77 | 18.6 (14.9-22.3) | 16,027 | 30.7 (30.3-31.1) |
| ≥ 30 | 337 | 81.4 (77.7-85.1) | 36,120 | 69.3 (68.9-69.7) |
| **Race** |  |  |  |  |
| Mixed | 219 | 52.9 (48.1-57.7) | 25,673 | 49.2 (48.8-49.7) |
| Non-mixed ^a^ | 195 ^b^ | 47.1 (42.3-51.9) | 26,474 | 50.8 (50.3-51.2) |
| **Schooling (full years)** |  |  |  |  |
| 0-4 | 229 | 55.3 (50.5-60.1) | 20,023 | 38.4 (38.0-38.8) |
| > 4 | 169 | 40.8 (36.1-45.6) | 26,228 | 50.3 (50.0-50.7) |
| Children/teenagers ^c^ | 16 | 3.9 (2.0-5.7) | 5,896 | 11.3 (11.0-11.6) |
| **Residential area** |  |  |  |  |
| Urban | 284 | 68.6 (64.1-73.1) | 36,393 | 69.8 (69.4-70.2) |
| Rural | 130 | 31.4 (26.9-35.9) | 15,754 | 30.2 (29.8-30.6) |
| **Total** | **414** | **100.0** | **52,147** | **100.0** |

^a^ White, black, Asian, or indigenous;

^b^ White = 139 (33.6%; 95% CI: 29.0-38.1); black = 51 (12.3%; 95% CI: 9.2-15.5); Asian = 3 (0.7%; 95% CI: 0.0-1.5) and indigenous = 2 (0.5%; 95% CI: 0.0-1.2);

^c^ Individuals aged < 18 years old.

CI: Confidence Interval.
