# Supplementary figures and images for: Leprosy and cutaneous leishmaniasis affecting the same individuals: A retrospective cohort analysis in a hyperendemic area in Brazil
Source: PLoS Negl Trop Dis. 2021 Dec 13;15(12):e0010035. doi: 10.1371/journal.pntd.0010035 (PMC8699965; doi:10.1371/journal.pntd.0010035)

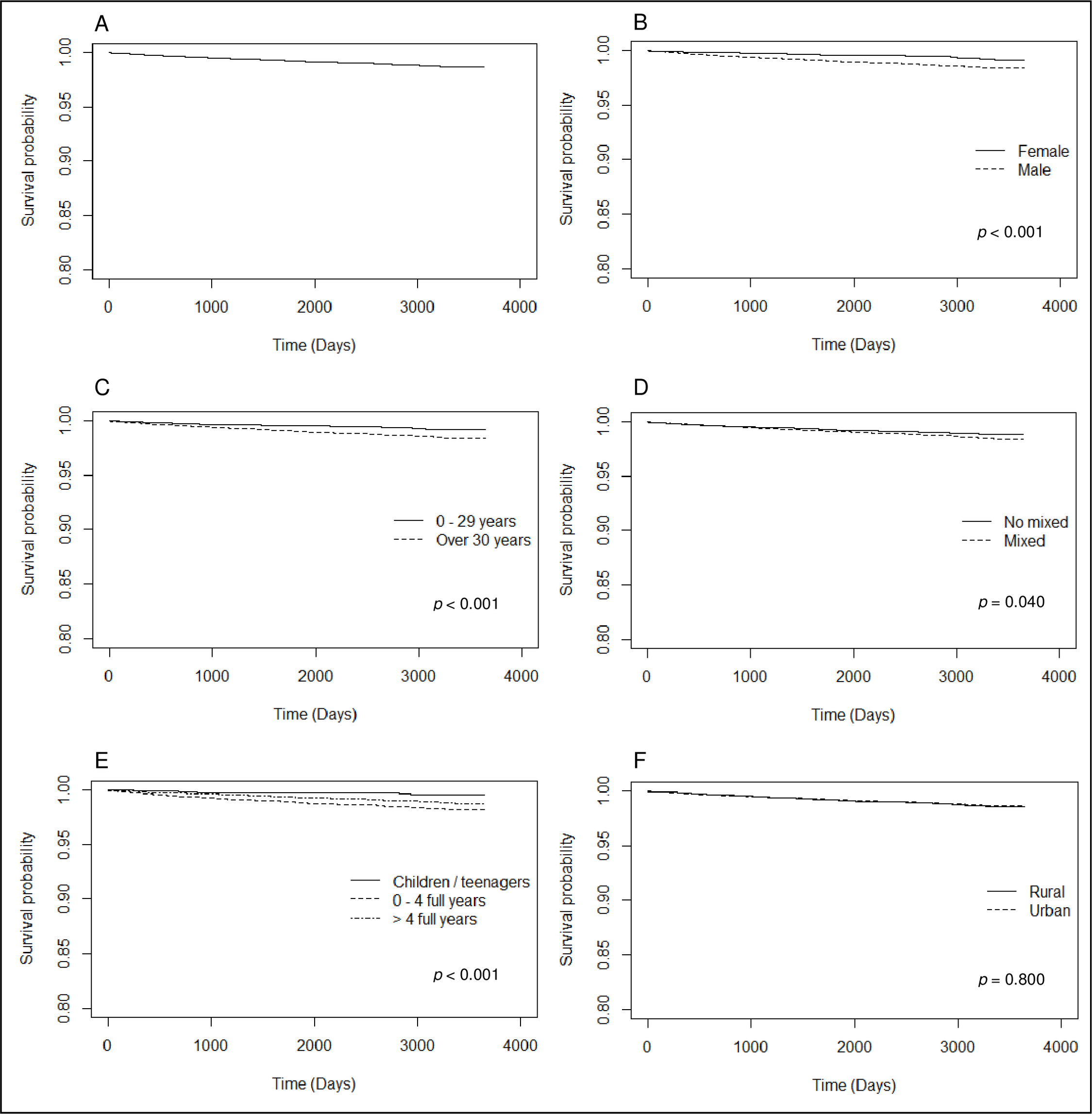

Supplement: S1 Fig — (A) Full cohort. (B) Sex. (C) Age group. (D) Race. (E) Schooling. (F) Residential area. The p values of the log-rank test were represented for each variable. Data from Mato Grosso state, Brazil, 2008–2017. (TIF) [file pntd.0010035.s003.tif]
