# Supplementary material for: Leprosy and cutaneous leishmaniasis affecting the same individuals: A retrospective cohort analysis in a hyperendemic area in Brazil
Source: PLoS Negl Trop Dis. 2021 Dec 13;15(12):e0010035. doi: 10.1371/journal.pntd.0010035 (PMC8699965; doi:10.1371/journal.pntd.0010035)
Supplement: S1 STROBE Checklist — (DOCX) [file pntd.0010035.s004.docx]

STROBE Statement—Checklist of items that should be included in reports of ***cohort studies***

|  | Item No | Recommendation |
| --- | --- | --- |
| **Title and abstract** | 1 | (*a*) Indicate the study’s design with a commonly used term in the title or the abstract – *Page 1 (“Title”): “Leprosy and cutaneous leishmaniasis affecting the same individuals: a retrospective cohort analysis in a hyperendemic area in Brazil” and Page 3 (“Abstract”- Lines: 58-59).* |
|  |  | (*b*) Provide in the abstract an informative and balanced summary of what was done and what was found – *Page 3 (Lines: 58-69).* |
| Introduction | | |
| Background/rationale | 2 | Explain the scientific background *(Pages 5-6)* and rationale for the investigation being reported – *Page 6 (Lines: 128-130).* |
| Objectives | 3 | State specific objectives, including any prespecified hypotheses – *Page 6 (Lines: 130-133).* |
| Methods | | |
| Study design | 4 | Present key elements of study design early in the paper – *Page 7 (“Study design”).* |
| Setting | 5 | Describe the setting, locations, and relevant dates, including periods of recruitment, exposure, follow-up, and data collection – *Pages 6-7 (“Study setting” and Lines: 150-152).* |
| Participants | 6 | (*a*) Give the eligibility criteria, and the sources and methods of selection of participants – *Page 8 (Lines: 167-173) and Fig 1A.*  (b) For matched studies, give matching criteria and number of exposed and unexposed – *No applicable.* |
| Variables | 7 | Clearly define all outcomes, exposures, predictors, potential confounders, and effect modifiers. Give diagnostic criteria, if applicable – *Pages 9-10 (Lines: 208-210*; *Pages 11-12 (Lines:249-253; 263-264; 278-280).* |
| Data sources/ measurement | 8 | For each variable of interest, give sources of data and details of methods of assessment (measurement). Describe comparability of assessment methods if there is more than one group – *Pages 7-13 (“Data collection and study population” and Lines:198-202; 220-224; 249-253; 262-269).* |
| Bias | 9 | Describe any efforts to address potential sources of bias – *S1 Table.* |
| Study size | 10 | Explain how the study size was arrived at – *No applicable.* |
| Quantitative variables | 11 | Explain how quantitative variables were handled in the analyses. If applicable, describe which groupings were chosen and why – *Page 12 (Lines: 270-277).* |
| Statistical methods | 12 | (*a*) Describe all statistical methods, including those used to control for confounding – *Pages 10-13 (“Spatial distribution” and “Survival analysis and Cox regression”).* |
|  |  | (*b*) Describe any methods used to examine subgroups *(No applicable)* and interactions – *Page 12 (Lines: 263-269).* |
|  |  | (*c*) Explain how missing data were addressed – *Page 12 (Lines: 257-260).* |
|  |  | (d) If applicable, explain how loss to follow-up was addressed – *No applicable.* |
|  |  | (*e*) Describe any sensitivity analyses – *S1 Table.* |
| Results | | |
| Participants | 13* | (a) Report numbers of individuals at each stage of study—eg numbers potentially eligible, examined for eligibility, confirmed eligible, included in the study, completing follow-up, and analysed – *Page 13 (Lines: 297-302) and Figs 1A and 1B.* |
|  |  | (b) Give reasons for non-participation at each stage – *No applicable.* |
|  |  | (c) Consider use of a flow diagram – *Fig 1.* |
| Descriptive data | 14* | (a) Give characteristics of study participants (eg demographic, clinical, social) and information on exposures and potential confounders – *Page 15 (Lines: 325-329) and S2 Table.* |
|  |  | (b) Indicate number of participants with missing data for each variable of interest – *No applicable.* |
|  |  | (c) Summarise follow-up time (eg, average and total amount) – *Page 13 (Lines: 297-299).* |
| Outcome data | 15* | Report numbers of outcome events or summary measures – *Page 16 (Lines: 344-350) and S1 Fig.* |
| Main results | 16 | (*a*) Give unadjusted estimates and, if applicable, confounder-adjusted estimates and their precision (eg, 95% confidence interval). Make clear which confounders were adjusted for and why they were included – *Page* *16 (Lines: 351-360) and Table 2.* |
|  |  | (*b*) Report category boundaries when continuous variables were categorized – *Page 16 (Lines: 357-360).* |
|  |  | (*c*) If relevant, consider translating estimates of relative risk into absolute risk for a meaningful time period – *No applicable.* |
| Other analyses | 17 | Report other analyses done—eg analyses of subgroups and interactions, and sensitivity analyses – *Pages 13-14 (Lines: 302-310) and Table 1. Page 15 (Lines: 329-336) and Fig 2. Page 16 (Lines: 360-364) and Fig 3.* |
| Discussion | | |
| Key results | 18 | Summarise key results with reference to study objectives – *Page 18 (Lines: 384-389).* |
| Limitations | 19 | Discuss limitations of the study, taking into account sources of potential bias or imprecision. Discuss both direction and magnitude of any potential bias – *Pages 20-21 (Lines: 451-464).* |
| Interpretation | 20 | Give a cautious overall interpretation of results considering objectives, limitations, multiplicity of analyses, results from similar studies, and other relevant evidence – *Pages 17-21 (Discussion).* |
| Generalisability | 21 | Discuss the generalisability (external validity) of the study results – *Page 21 (Lines: 465-475).* |
| Other information | | |
| Funding | 22 | Give the source of funding and the role of the funders for the present study and, if applicable, for the original study on which the present article is based – *Brazilian National Council for Scientific and Technological Development (CNPq), process number 421138/2018-1* – *project founder.* |

*Give information separately for exposed and unexposed groups.

**Note:** An Explanation and Elaboration article discusses each checklist item and gives methodological background and published examples of transparent reporting. The STROBE checklist is best used in conjunction with this article (freely available on the Web sites of PLoS Medicine at http://www.plosmedicine.org/, Annals of Internal Medicine at http://www.annals.org/, and Epidemiology at http://www.epidem.com/). Information on the STROBE Initiative is available at www.strobe-statement.org.
